# Calculation of Measurement Uncertainty Using Prior Information

**DOI:** 10.6028/jres.103.042

**Published:** 1998-12-01

**Authors:** S. D. Phillips, W. T. Estler, M. S. Levenson, K. R. Eberhardt

**Affiliations:** National Institute of Standards and Technology, Gaithersburg, MD 20899-0001

**Keywords:** Bayesian, bias, error, measurement uncertainty, uncertainty

## Abstract

We describe the use of Bayesian inference to include prior information about the value of the measurand in the calculation of measurement uncertainty. Typical examples show this can, in effect, reduce the expanded uncertainty by up to 85 %. The application of the Bayesian approach to proving workpiece conformance to specification (as given by international standard ISO 14253-1) is presented and a procedure for increasing the conformance zone by modifying the expanded uncertainty guard bands is discussed.

## 1. Introduction

**Fig. 1:**
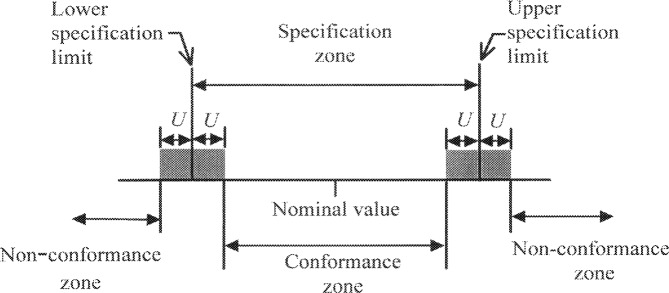
A typical functional specification of a workpiece and the corresponding inspection zones; *U* is the expanded uncertainty of the measurement. Workpieces are accepted if the measurement result is within the conformance zone.

The ISO *Guide to the Expression of Uncertainty in Measurement* (the *Guide*) [1], and the associated NIST adaptation [2], have described a unified convention for expressing measurement uncertainty. Application of the *Guide* has extended beyond calibration and research laboratories and into the industrial domain of manufactured products. Recently, international standard ISO 14253-1 *Inspection by measurement of workpieces and measuring instruments—Part 1: Decision rules for proving conformance or non-conformance with specification* [3], has explicitly included measurement uncertainty in proving conformance of products. These decision rules reward accurate metrology by increasing the conformance zone commensurate with the decreases in measurement uncertainty, as illustrated in Fig. 1. In this procedure the measurement uncertainty is defined as the expanded uncertainty of the result of the measurement, denoted *U*, with a default coverage factor of *k* = 2. This technique is appropriate if no prior measurement information is known about the workpiece under inspection. However, if prior measurement information which appropriately characterizes the population of this type of workpiece is available, for example, from historical inspection records, then this information may be used to further increase the conformance zone. This can be of significant economic benefit to manufacturers who maintain historical records of their measurement results. While the use of this principle, which involves Bayesian inference, is well known in the statistical community, it is generally unknown to metrologists. It is the purpose of this paper to describe this Bayesian approach with emphasis on the application to acceptance and rejection of workpieces based on the 14253-1 decision rules. The paper applies the approach to two examples and gives practical advice on the use of the approach as well.

## 2. Bayesian Approach

Bayesian inference provides a rigorous means of incorporating prior information into a measurement. It is based on the mathematics of conditional probability distributions. Although it is beyond the scope of this paper to review Bayesian inference, the central result we will employ is given in Eqs. (1) and (2), which assumes that a Gaussian distribution is descriptive of both the measurement and workpiece uncertainties [4]. Furthermore, it is crucial that the distribution embodied by the prior information be applicable to the workpiece currently being measured (this is further discussed in Sec. 6).

$$y=y_{m}\left(\frac{\gamma^{2}}{1+\gamma^{2}}\right)+y_{\mathrm{pe}}\left(\frac{1}{1+\gamma^{2}}\right)$$ (1)

where

$$\gamma =\frac{u_{\mathrm{pe}}}{u_{\mathrm{cm}}}$$

$$\frac{1}{u_{c}^{2}}=\frac{1}{u_{\mathrm{cm}}^{2}}+\frac{1}{u_{\mathrm{pe}}^{2}},$$ (2)

and *y* is the best estimate of the measurand (the quantity subject to measurement) including prior information.

*y*_m_: is the best estimate of the measurand without including prior information; this would be the measurement result without Bayesian analysis.
*y*_pe_: is the best estimate of the measurand based on prior (historical) information of the workpiece population; this is usually close, but in general not equal to, the target specification value, i.e., the nominal value.
*u*_c_: is the combined standard uncertainty associated with the measurement when prior information is included.
*u*_cm_: is the combined standard uncertainty of the measurement without using prior information; this includes not only the instrumentation uncertainty but also that due to the environment, operator, and other factors affecting the measurement value.
*u*_pe_: is the standard deviation of the probability distribution which describes the measurand based on prior (historical) information.

It is clear from Eqs. (1) and (2) that two effects occur when prior information is included in the measurement analysis. Firstly, the best estimate of the measurand is shifted from *y*_m_ toward *y*_pe_. Secondly, the combined standard uncertainty *u*_c_ decreases due to the use of prior information. Equation (1) can be interpreted as stating that the best estimate of the measurand is a weighted average of the estimate from the measurement and that from all prior information about the measurand. Similar comments pertain to Eq. (2), which gives the combined standard uncertainty; in particular, note that the uncertainty is always less than *u*_cm_, i.e., the use of prior information can only decrease the uncertainty associated with the measurand and will never increase it.

We now present two exceptional but informative examples to illustrate the Bayesian approach. Consider the case of measuring a characteristic of a workpiece whose value is almost exactly known from previous measurements. This might be typical of measuring a calibrated gauge block with a hand-held caliper (having a combined standard uncertainty of 10 μm). The length distribution of good quality manufactured (short) gauge blocks is very narrow (typically having a standard deviation less than 0.05 μm), and the calibration uncertainty is even smaller; thus *γ* << 1. Equation (1) then yields *y* ≈ *y*_pe_, and Eq. (2) yields *u*_c_ ≈ *u*_pe_, i.e., regardless of the measuring instrument (caliper) result, the best estimate of the gauge block length is *y*_pe_, with uncertainty *u*_pe_. Thus the best estimate of the block’s length is based on the (accurate) historical mean value not the current (inaccurate) measurement result, agreeing with common sense.

Our second example considers very accurate measurements of workpieces which come from a very broad production distribution. In this case (because of the broad workpiece distribution), *u*_pe_ >> *u*_cm_, so *γ* → ∝, then Eq. (1) reduces to *y* ≈ *y*_m_, and Eq. (2) reduces to *u*_c_ ≈ *u*_cm_, stating that the best estimate of the measurand and its uncertainty is just the value from the measurement result; this also agrees with common sense.

## 3. Application to Decision Rules

Equations (1) and (2) are based on Bayesian inference and are independent of any particular decision rule application. Applying this method to the particular decision rule given by 14253-1 is best illustrated by presenting some examples. For illustrative purposes in these examples we will assume that the process is well under control and there exist ample historical data. In later sections, we examine these assumptions.

For application purposes it is often convenient to consider the amount of the Bayesian adjustment to the measurement result, Δ*y*, and also the amount of adjustment to the expanded uncertainty Δ*U*, given by

$$\Delta y=\left|y_{m}-y\right|=\frac{\left|y_{m}-y_{\mathrm{pe}}\right|}{1+\gamma^{2}}$$ (3a)

$$\Delta U=U_{m}-U_{B}=U_{m}\left(1-\sqrt{\frac{\gamma^{2}}{\gamma^{2}+1}}\right).$$ (3b)

Here *U*_m_ = 2 *u*_cm_ is the unadjusted expanded uncertainty, and *U*_B_ = 2 *u*_c_, is the expanded uncertainty including the Bayesian adjustment.

**Fig. 2:**
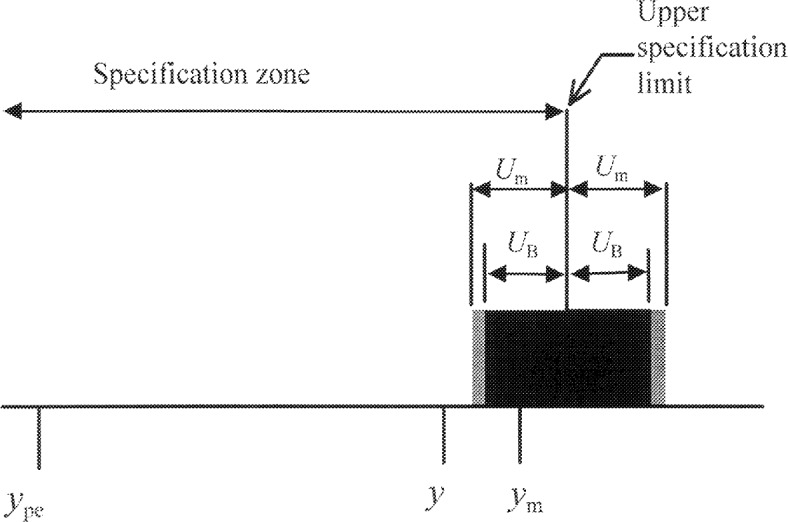
Illustration showing the expanded uncertainty without Bayesian adjustment *U*_m_, and with Bayesian adjustment *U*_B_, together with the best estimate of the measurand *y*, using Bayesian inference.

First we consider a typical case of gauge recalibration. Suppose for a particular type of workpiece (e.g., gauge), the historical records show that 95 % of the measurement results of the gauges returned for periodic recalibration are within the specification zone and that the distribution is approximately Gaussian. (In dimensional metrology this means that 95 % of the workpieces have a measured value within the permissible workpiece tolerance.) The mean value of the historical data is *y*_pe_, which we assume to be in the center of the specification zone. The best estimate of the dispersion of the measurand, given by the standard deviation of the historical measurements, is *u*_pe_. Suppose further that the inspection process uses a 4:1 gauging ratio, i.e., 2 *U*_m_ = (1/4) *T*, where *U*_m_ = 2 *u*_cm_ and *T* is the workpiece tolerance, i.e., the width of the specification zone, and *u*_cm_ is the combined standard uncertainty of the measuring instrument. Hence *u*_cm_ = *T*/16. Now to calculate *u*_pe_ we must deconvolve the measurement uncertainty; therefore *u*_pe_ = [(1/4)^2^ − (1/16)^2^]^1/2^ $T=(\sqrt{15}/16)$ *T*. Consequently, $\gamma =\sqrt{15}$. If a workpiece (produced from the same parent distribution as the historical data) is inspected and yields a measured value of *y*_m_, then the best estimate of the measurand is *y* = (15/16) *y*_m_ + (1/16) *y*_pe_. Figure 2 is a schematic diagram of this example; it shows that the best estimated expanded uncertainty *U*_B_ (where *U*_B_ = 2 *u*_c_), is slightly smaller than *U*_m_ (the *γ* → ∝ case), and that the best estimate of the measurand is shifted toward the mean value (*y*_pe_) of the historical workpiece distribution.

From this example it is clear that the only cases of consequence are measurement values near the specification limit, i.e., a value well outside the specification zone will continue to be in the non-conformance zone even after the application of the Bayesian method.

Using the values assumed in the above example, we can calculate the combined effect of the Bayesian adjustments to the measurement result and the expanded uncertainty. The largest measurement result (*y*_m_), that will prove conformance after the Bayesian adjustment is implicitly given by the equality *y = T*/2 − *U*_B_, where *y* and *U*_B_ are the best estimate of the measurand and expanded uncertainty after the Bayesian correction. (For convenience we have centered the specification zone around zero, and hence *y*_pe_ is also zero.) Substituting the definitions of *y* and *U*_B_ (where *U*_B_ = 2 *u*_c_) given by Eqs. (1) and (2) determines the largest value of *y*_m_ that will prove conformance after the adjustment. In this example, the effective conformance zone is increased by 7.8 %. Equivalently, the situation can be viewed as using a measuring instrument having only 77 % of the uncertainty compared to the no Bayesian adjustment case; either interpretation shows that the use of prior information can have a significant effect.

A similar example typical of precision workpiece production may have 99 % of the workpieces historically in the specification zone when using a gauging ratio of 3:1, implying that 2 *U*_m_ = (1/3) *T*, hence *u*_pe_ = [(1/6)^2^ − (1/12)^2^]^1/2^ $T=(\sqrt{3}/12)$ *T* with $\gamma =\sqrt{3}$.

In this example the conformance zone is increased by 42 %, or equivalently using a measuring instrument having only 15.5 % of the uncertainty compared to the no Bayesian adjustment case. As these examples show, the significance of prior information in the uncertainty calculation greatly increases as the ratio of *u*_pe_: *u*_cm_ = *γ* decreases.

## 4. Treatment as a Pseudo-Bias

**Fig. 3:**
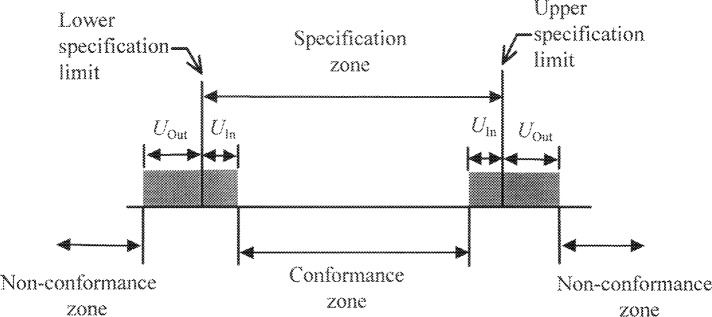
Modification of the expanded uncertainty to account for prior information about the workpiece under inspection.

The procedure outlined above describes how each measurement result can be corrected using prior information. This correction is similar to that performed for a systematic error, i.e., bias. However, it has the unusual property that measurement results above the mean are decreased (as would be the case for a positive bias) whereas measurement results below the mean are increased (as would be the case for a negative bias). In some situations it may be desirable to account for a bias by modifying the quoted expanded uncertainty rather than correcting each measurement result individually [5]. This can be accomplished by defining *U*_In_ and *U*_Out_ as shown in Fig. 3 and given by Eqs. (4a) and (4b), where we require *U*_In_ to be ≥ 0 in Eq. (4a), i.e., if *U*_In_ becomes negative we let *U*_In_ = 0. Equations (4a) and (4b) require the value of Δ*y* which depends on the difference (*y*_m_ − *y*_pe_) as given in Eq. (3a). A reasonable approximation can be obtained by substituting *T*/2 for (*y*_m_ − *y*_pe_). The exact values can be determined by solving implicit equalities similar to what was described in Sec. 3:

$$U_{\mathrm{In}}=U_{B}-\Delta y$$ (4a)

and

$$U_{\mathrm{Out}}=U_{B}-\Delta y$$ (4b)

The use of the pseudo-bias approach produces results similar to correcting each individual measurement using Eqs. (1) and (2), i.e., more workpiece measurement results lie in the (increased) conformance zone and fewer lie in the non-conformance zone. If we have no prior information about the measurand, i.e., *γ* → ∝, then *U*_In_ = *U*_Out_ = *U*_m_ and Fig. 3 reverts to Fig. 1. If we have very accurate information about the mean of the workpiece distribution and we know that the distribution is narrow (this was the case of the previously described example of measuring a calibrated gauge block with a hand held caliper), then *γ* is small and (by using the requirement of non-negative *U*_In_) *U*_In_ = 0, i.e., the conformance zone equals the specification zone. In this rather extreme situation the pseudo-bias approach (conservatively) prevents proving conformance of a workpiece if the measurement result lies outside the specification zone, whereas using Eq. (1) the Bayesian correction may be sufficiently large to shift the measurement result inside the conformance zone. However, the situation where *U*_In_ = 0 is an indication of poor metrology and that the measuring process should have its uncertainty reduced.

## 5. Example Sensitivity and Cost Analysis

Bayesian inference provides a natural and consistent way to make best use of all relevant information. Its effectiveness depends to a large degree on the reliability of the information. In this section, we consider the cost issues associated with using decision rule 14253-1, both with and without the Bayesian adjustment, and we also investigate the sensitivity of the costs to the reliability of the available information.

There are two costs that should be considered when evaluating a workpiece acceptance rule. There is the cost of accepting a bad workpiece (Type I cost) and the cost of rejecting a good workpiece (Type II cost). The cost of accepting a bad workpiece may include the cost of a malfunctioning product, the cost of replacing the workpiece, etc. If a good workpiece is rejected, the cost might be the cost of reworking the workpiece unnecessarily or the cost of the production of the workpiece itself. We will give emphasis to the case in which the Type I cost is larger than the Type II cost. In addition to cost considerations, it is important to consider the percentage of good workpieces versus bad workpieces in the population. For example, if the percentage of bad workpieces is small relative to that of good workpieces, then the Type I cost is less important than in the case in which the percentage is large.

Based on certain probabilities that are described below, it is possible to determine the expected cost of using a given decision rule. If the Type I and Type II costs are expressed on a per workpiece basis, the expected cost gives the cost per workpiece of using the rule. Since it is impossible to make perfect decisions, the expected cost is always positive. Mathematically, the expectation is equal to *αC*_1_ + *βC*_2_, where *α* is the probability that the workpiece is bad AND the workpiece is accepted; *β* is the probability that the workpiece is good AND the workpiece is rejected; *C*_1_ is the Type I cost per workpiece, and *C*_2_ is the Type II cost per workpiece. We consider a workpiece to be “good” if the value of the measurand lies within the specification zone and “bad” if it lies outside the specification zone. We assume a workpiece is accepted if it is in the conformance zone and rejected otherwise.

To calculate the probabilities *α* and *β*, we need to know the true underlying distribution of workpieces, as well as a decision rule. Table 1 gives the *α* and *β* probabilities for the 14253-1 rule both with and without the Bayesian adjustment for the two examples of the Sec. 3. We have also included the cost of the decision rule (as a percentage of the unit workpiece cost) for the case where Type I costs are 15 times larger than Type II costs. For these calculations we assumed that the underlying population is a Gaussian distribution and centered in the conformance zone and with standard deviation *u*_pe_. This amounts to assuming that the prior distribution used in the Bayesian rule is the true underlying distribution. Also note that the Type I probabilities (alpha) are all less than 0.001. Such small probabilities are heavily dependent on the tail behavior of the Gaussian distribution, which might not be valid in the real world. However, these small probabilities do not significantly affect the cost estimates we present in Table 1.

In both examples *u*_pe_ is small relative to the conformance zone and large relative to *u*_cm_, which results in a small percentage of bad workpieces and decreased importance of the Bayesian adjustment. Nevertheless, the use of the Bayesian adjustment has a significant effect on the cost of using the decision rule. In example 1 there is a 22 % reduction in the cost of using the decision rule, and a 87 % cost reduction in example 2. Although these examples depend upon the specific values of *u*_cm_, *u*_pe_, *T*, and the ratio of Type I to Type II costs, the use of the Bayesian adjustment will generally have significant beneficial economic impact on implementing the decision rule.

In real-life applications of using a decision rule, one can only estimate the standard deviation of the prior distribution of workpieces *u*_pe_ and the uncertainty of the measurement system *u*_cm_. A rule may be very good if reliable information is available on these quantities, but may perform badly if the information is not reliable. A good rule in practice must be robust to reasonable errors in the estimation of these two standard deviations.

**Fig 4:**
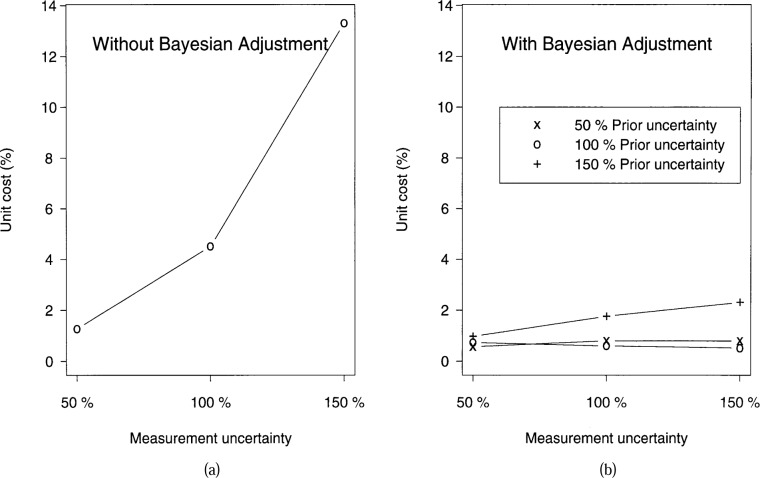
Sensitivity analysis of decision rule 14253-1 with and without the Bayesian adjustment showing the cost (as a percentage of unit workpiece cost) incurred when using the rule. (a) without the adjustment as a function of the accuracy in estimating *u*_cm_. (b) with the adjustment as a function of the accuracy in estimating both *u*_cm_ and *u*_pe_.

Using the framework of the second example, we examine the sensitivity of the 14253-1 rule, both with and without the Bayesian adjustment, to the accuracy of the estimation of *u*_pe_ and *u*_cm_. We assumed that the cost of accepting a bad workpiece is 15 times the cost of rejecting a good workpiece. Figures 4a and 4b summarize the effect on the cost of various estimates. The vertical axes give the expected cost per workpiece (as a percentage of workpiece cost) due to various errors in the estimation of the standard deviations. The percentages on the horizontal axes correspond to estimating *u*_cm_ to be 50 %, 100 %, and 150 % of its best evaluation, i.e., 100 % is the best evaluation of the measurement uncertainty. Likewise, the three lines in Fig. 4b correspond to evaluating *u*_pe_ by 50 %, 100 %, and 150 % of its best evaluation. Since the case of not using the Bayesian adjustment (shown in Fig. 4a), does not make use of *u*_pe_, only the sensitivity to *u*_cm_ is considered for it.

In this example, the use of the Bayesian adjustment is rather robust to the errors in the estimation of both *u*_pe_ and *u*_cm_. Additionally, for all three estimates of *u*_cm_, the Bayesian adjustment procedure has lower cost than not using the adjustment, independent of which of the three values of *u*_pe_ are used. Specifically, if the Bayesian adjustment is not employed, the decision rule is particularly sensitive to overestimation of *u*_cm_. These results depend on the values of *u*_pe_, *u*_cm_, and the ratio of the two costs; however, we have found similar trends using a variety of values.

## 6. Implementation Considerations

Central to the use of the Bayesian approach is that the historical information is characteristic of the workpiece under measurement. Hence, the production process must be reasonably stable and free from drift. For example, if the historical information is based on measurements performed in summer (and have not been corrected for thermal expansion), and the workpiece under consideration is produced in winter, the historical mean value *y*_pe_ may not represent the current production process mean. Since the prior distribution is not representative of the current production process, the Bayesian adjustment is inappropriate. Additionally, whenever |*y*_m_ − *y*_pe_| >> *u*_c_ it is highly probable that something in the production or measurement process (or both) has significantly changed and the source of this discrepancy should be investigated. Once the problem has been resolved one must reconsider whether the historical information still appropriately describes the current process. Standard practices of statistical quality control [6] may be employed to test the appropriateness of the historical information. In particular, control charts can detect changes in the mean, variations of the process, and short and long term drifts. Appropriate plots may reveal other departures such as batch effects.

Equations (1) and (2) are strictly valid when only the prior and measurement distributions are Gaussian. Although modifications to these equations exist for other distributions, they become increasingly complex. Processes which are not optimized either regarding the workpiece production distribution or the measurement distribution are usually not characterized by a Gaussian distribution since one or two factors often dominate the process. Histograms and Q-Q Plots [7] are useful diagnostics here.

In the examples of Sec. 3, we deconvolved the measurement uncertainty from the underlying workpiece distribution. In practice, using the measured workpiece distribution (which includes the measurement uncertainty) to determine *u*_pe_ is advisable since it avoids the consequences of poorly estimating *u*_cm_ while usually only slightly decreasing the magnitude of the Bayesian adjustment.

Finally, many process capability techniques, such as statistical process control and gauge repeatability and reproducibility studies, often measure only the variation in the process and do not provide accurate information on the mean value. The use of calibrated artifacts or measuring equipment, which yields an estimate of the measurand, as well as the measurement variation, should be used to ascertain the historic mean value *y*_pe_. Some processes are deliberately biased toward the specification limits; for example, internal diameters may be produced to have an average value close to the lower specification limit since reworking a workpiece can always remove material, but cannot replace it. In such situations the benefit of the Bayesian adjustment will be reduced since the historic mean value *y*_pe_ and the measurement result *y*_m_ will generally be closer together.

## 7. Summary

We have described the use of Bayesian inference to include prior information in measurement uncertainty calculations. This procedure, when combined with the 14253-1 decision rules, can result in a significant increase in the size of the conformance zone. One simple technique of implementing the Bayesian method is to adjust the expanded uncertainty guard bands which determine the conformance zone. A sample cost sensitivity analysis demonstrated a cost savings when using the Bayesian adjustment with the 14253-1 decision rule.

## Figures and Tables

**Table 1:** Probability of Type I errors (*α*) and Type II errors (*β*) and the associated costs of using the 14253-1 decision rule both with and without Bayesian corrections, we assume Type I costs: Type II costs are 15:1

14253-1 decision rule	% bad workpieces	*α*	*β*	Cost of using rule as percentage of cost of workpiece (%)
Example 1 without Bayesian correction	3.9	0.000175	0.0949	9.75
Example 1 with Bayesian correction	3.9	0.000548	0.0676	7.58
Example 2 without Bayesian correction	0.053	0.000006	0.0450	4.51
Example 2 with Bayesian correction	0.053	0.000131	0.00404	0.60
